# Edinburgh Hospital Reports

**Published:** 1894-03

**Authors:** 


*Edinburgh Hospital Reports.* Edited by G. A. Gibson, M.D.; C. W. Cathcart, M.B.; John Thomson, M.D.; D. Berry Hart, M.D. Volume I. Pp. xiv., 620, 56. Edinburgh: Young J. Pentland. 1893.

When we think of the great part the Edinburgh Infirmary has played in educating generations of medical men, and the prestige it has gained from the traditions inherited from the long series of men, pre-eminent in medicine and surgery, who have served on its staff, it seems somewhat surprising that a hospital of such importance should have hitherto issued no official reports. The editors tell us however that there were difficulties in the way, which have only lately been removed, and now we have a volume representative of the work of three of the Edinburgh hospitals. The medical world will read these Reports with interest, because they will reflect from year to year the character of the work and teaching of the leaders of medical thought in Edinburgh.

A large body of old students must look to their Alma Mater for enlightenment and help, and will expect in these Reports to find records of the progress made in Medicine and Surgery. Nor will they be disappointed if future volumes—and there is no reason to doubt that they will do so—furnish so able and interesting a collection of papers as is gathered together in this first number. It opens, as is fitting in the first of a series, with a short history of the institutions whose work is to be chronicled. We cannot attempt here even to enumerate the number of excellent articles in this book, all of which will repay perusal, and we therefore must content ourselves with cordially recommending it to our readers as a volume worthy of careful study.

We feel sure that the editors will have no reason to regret the issue of these Annual Reports, and that they will have the support of a wide circle of readers.

